# Foreign

**Published:** 1841-05-29

**Authors:** 


### FOREIGN.

Our last files of foreign journals are filled with animated discussions, and reports of the transactions of medical societies, in relation to the several new operations proposed for the cure of stammering. The operations are those of Yearsley, Lucas, and Dieffenbach: each founded upon a different principle, and yet each professing to accomplish a cure in the majority of cases. Mr. Yearsley gives the following account of the manner in which he was led to the discovery of the operation which he recommends:

In the practice of my department of the profession, it has been usual with me to explore the condition of the mouth and pharynx in every case of deafness committed to my care. I have thus discovered that a large number of patients suffering from deafness are affected with enlargement of the tonsils and uvula, and an irritable condition of their investing membrane and the pharynx generally. It has been my constant practice, when I have considered these states at all contributing to the imperfection in hearing, to remove either the tonsils or the uvula, or portions of both, according to the nature of the case, with the most marked and immediate benefit, as far as the hearing may have been concerned. In December last it occurred to me to operate in this manner on two patients. They were, at the time of treatment, so deaf, that I did not then address my questions particularly to them, but to their parents, so that I was unaware of any impediment to speech in these instances. Some time after, as the cure of deafness advanced, I learned from the parents that both children had been stammerers from infancy, and, as much to my surprise as gratification, that the cure of stammering had ensued immediately on the excision of the tonsils. At the time at which I write, the subjects of both these cases remain free from any impediment, though their stammering previously to the operation is represented to me as having been very decided. I had before this remarked that persons with enlarged tonsils were affected with thick and imperfect speech, for which I had often, during the last year, practised excision with the happiest effect, in restoring the voice to its original clearness. Since the cases above mentioned, I have operated on upwards of forty persons, all of whom have immediately felt themselves relieved of their impediment. Many have seemed wild with joy, or have shed tears of pleasure, at the instantaneous restoration they have enjoyed. After the operation, the difficulty of speech which remains is referred by the patient to the lips; they express themselves entirely free from the original difficulty. Something must be allowed for the long misuse of the organ of voice, and the existence of habit, in rendering the voice less perfect than in the natural state. In fact, after their relief, patients have yet to learn the proper use of the vocal apparatus.

I have performed the operation by means of a scalpel, tenaculum, and scissors, without any serious hæmorrhage, and with a small amount of pain, which has appeared somewhat greater in the case of the uvula than the tonsils.

In reflecting upon the subject, the explanation I have at present to offer is, that to produce stammering, the dorsum linguæ, the palatine arches, velum palati, and uvula, approximate together so completely, and perhaps irregularly, as to leave no room for the expulsion of air from the larynx. In a person who stammers, no air issues from the mouth during the abortive effort to speak; but it does so as soon as the patient is relieved from this state, so as to produce sound. The most violent contractions of the abdominal muscles can be seen attempting to force up the diaphragm and expel the air; sometimes all the respiratory muscles, and even those of the body generally, are thrown into violent spasmodic action, as the individual grasps some near object to assist the expulsive effort. In some cases, when there is nothing abnormal about the tonsils or uvula, I find a great congenital narrowing of the entrance from the mouth to the pharynx.

Mr. Yearsley, it appears, has operated upon one hundred and twenty patients, of whom only three were women! the great majority of these were cured, all were more or less relieved—so say Mr. Yearsley's partisans. The London Medical Society, however, openly condemns every operation, because "stammering is essentially functional," and the operations in question "are not based on any scientific basis." While the Westminister Medical Society admitting, what certainly cannot be disputed, that real cures have *occasionally* followed these attempts—particularly those of Mr. Yearsley, which are the most numerous,—starts from this point as a fact, and occupies itself more usefully in endeavouring to trace out the relation between cause and effect. The following is a brief summary of the opinions expressed by some of the distinguished members in reference to the mode of action of the operation, condensed from the Lancet.

With regard to the extent of the operation, Mr. Yearsley, in most cases, removed the uvula entirely, chiefly with the view of throwing both arches of the palate into one. When the tonsils were so much enlarged as to project beyond the columns of the fauces, the uvula was then partially removed, and also so much of the tonsils as projected; in only one case had any thing like severe hæmorrhage took place, and in this the bleeding continued for four hours. The operation, however, might not be unattended by danger, when there was a diathesis to hæmorrhage present in the system.

Mr. Dowing, who had witnessed several of the operations, and was struck with the fact that three methods so entirely different in their character were all followed in the majority of cases by success, was disposed to consider the effect dependent on the shock to the nervous system, and the loss of blood.

Mr. Alcock also had seen many operations performed by Mr. Yearsley. Some of these were attended with *marvellous improvement*, others with great relief, while others were productive of no benefit—believing that stammering depended on a variety of causes, he could not conceive that Mr. Yearsley's method would be applicable to all cases, but had no doubt that in many it would be followed by a permanent cure.

Mr. Malyn had been present at three operations, all of which were successful, although, only in one instance, did the uvula upon inspection appear unnaturally elongated. Mr. Malyn believes that stammerers experience the greatest difficulty in uttering the labial or dental sounds, because in the effort the velum descends and hangs over the windpipe, thus preventing a sufficient supply of "material for the manufacture of words;" by the operation of Mr. Yearsley this impediment is removed, the supply of material is rendered sufficient, and the stammer ceases.

The opinion of Dr. Marshall Hall is more at length and more scientific; we quote it in his own words:

He observed, that the previous speakers had not, he thought, entered into the true line of argument. Too much had been said about the tumidity of the tonsils, and of the tongue; too little about the functional properties of the organs of articulation. Now, tumidity of the tonsils induced a certain well-known and easily recognisable thickness of the voice; tumidity of the tongue induced a special defect of articulation, but neither of these induced stammering. On the other hand, stammering was excited in a little patient of his whenever the general health was deranged. Dr. Bostock had detailed, in the "Medico-Chirurgical Transactions," a case of stammering cured by the administration of purgative medicines; and, lastly, stammering was excited as a part of chorea. These, and other facts, proved that stammering was not so much an organic as a functional defect; and the question to be agitated that evening was, what relation the excision of the uvula could have with the cure of such a malady. Dr. Hall had been witness to two cases: the first was that of Philip Wyatt; before the operation, which consisted in the removal of the uvula, the patient was asked his name; in vain he attempted to enunciate the Ph; the effort seemed to threaten convulsions; the operation was performed; the same question was put, and the ready reply was, Philip Wyatt! In the second case the good effect, though less complete, was not less obvious. Now, what could be the rationale of this phenomenon? That it could be at all connected with the more or less open state of the air-passages, Dr. Hall regards as most improbable. In many cases there was no enlargement of the tonsils or tongue, no elongation of the uvula, neither was there any want of volume or force of the expired air, when the word was well pronounced, or in the pronunciation of such letters as did not absolutely interrupt the flow of the expired air, as v., s., &c.; nor was there, as Dr. Arnott thought, any obstruction to the flow of air in the larynx itself, as he (Dr. Hall) had shown in a paper published in the "Journal of the Royal Institution," in 1831. The obstruction was offered by the organs, not of the voice, but of the articulation; not of the physical condition of the parts, but of their undue action. In pronouncing the letter b, the mouth was closed by the force on adduction of the lips, the posterior nares being closed by the veil of the palate. Long ago Dr. Hall had described stammering as an undue spinal action; the stammering of chorea proved this. He would now venture to ask, might the situation of the uvula and its peculiar contact with the parts of the posterior nares lead to a reflex spinal action? The act of vomiting on tickling the fauces was not less marvellous or inscrutaable; the uvula in this manner might be the excitor and regulator of speech. Stammering might be induced in cases in which its posterior surface was unduly excitable. In this manner we might explain the effects of Mr. Yearsley's operation; but Dr. Hall begged the society to view his last observation as a conjecture. Elongation of the uvula could have no effect in inducing stammering as supposed, by falling on the tongue; for in the enunciation of many letters, as b, t, v, s, &c., it was raised, with the velum, high up, so as to assist in closing the posterior nares. Time and further investigation were, as Mr. Yearsley was aware, required to mature the investigation, to determine the special cases of stammering to which the operation was adapted. But enough had been done to excite the deepest interest in every liberal mind.

Mr. Robins had witnessed several operations by Mr. Y., attended by such success as would induce him, although he believed stammering to depend on a variety of causes, to recommend to every one afflicted with this defect to submit to the removal of his uvula.

In a word, the conclusions to be drawn from the discussion before the Westminster Medical Society are as before stated. That the operations are occasionally followed by an entire and permanent cure, more frequently are attended by relief—the relief consisting in the removal of the spasm by which the stammer is accompanied, rather than in any benefit to the stammer—while in other cases the operation effects no change.

A question of preference however exists between the operation which we have just noticed and that recommended by Messrs. Lucas and Amusat. This latter consists in a division of the frœnum, and a removal of a portion of the anterior fibres of the genio-hyoglossi muscles; the relative advantages of these two methods, and the particular cases to which each is applicable, remain yet unsettled.

In order to complete the history of this subject as at present before the profession, we have only to introduce the more dangerous and difficult operations devised by Dieffenbach, which we extract in the words of his translator, Mr. Travers.

"The idea lately suggested itself to me," says Dieffenbach, "that an incision carried completely through the root of the tongue might possibly be useful" in stuttering, which had resisted other means of cure, "by producing an alteration in the condition of its nervous influence, allaying the spasm of the chordæ vocales, &c." A most unphilosophical beginning, truly, and one which speaks strongly of the discipline of the German hospitals, and possibly of the little danger of an inquest on unsuccessful practice. One thing is certain, that an idea so conceived would be regarded as most unwarrantable in its application in the British hospitals. Nor is the case improved, when he explains that the first thought of the operation originated in a patient with strabismus one day requesting to be operated on for that deformity, with a "well-marked stutter." But he continues—"the brilliant success of this new operation more than realized my most sanguine expectations."

The principle which the professor has in view, is the division of the nervous filaments distributed to the muscular substance of the tongue, under the impression that some undue excitation in these nerves induces the spasmodic movement of the muscles. That this effect may result from the operation is more than probable; but it appears to us that the method of arriving at the end in view is exceedingly clumsy. If mere alteration of the innervation of the muscles of the organ be the object, why not, by a much more simple, incomparably less severe, and less dangerous operation, at once divide the hypoglossal nerve or nerves as they rest on the hyo-glossus muscles; an operation requiring a superficial incision of scarcely an inch in length.

The author prescribes three methods of operating. "1. The transverse horizontal division of the root of the tongue. 2. The subcutaneous transverse division, in which the mucous covering of the tongue is left inviolate. 3. The horizontal division with excision of a wedge-shaped portion."

"The first operation I performed on the 7th of January, 1841. I chose for this case the method by which a wedge-shaped portion is removed from the posterior part of the tongue; for, as I have remarked, I felt more confidence in this than in the other methods.

"Frederick Doenau, a highly intelligent and talented boy of thirteen years of age, had stuttered from his earliest childhood, and to so painful an extent, that the defect was thought to be quite incurable. It varied, however, much in degree: when at the worst, he was unable even to produce a sound. He stuttered in Latin and French, as well as in his own language—sometimes on one set of words, and sometimes on others. The pronunciation of the sibilant letters (s, z, ss,) and of the palatals hard (g, k, ch, and x,) was attended with particular difficulty; and he made no distinction between the hard sounds, p, t, k, and the soft ones, b, d, g, (German.) He repeated the same letter often four times running; and when he whispered, he stuttered as much as when he spoke loud or shouted; often he could either not speak at all, or produced only half articulate sounds. The presence of a stranger invariably affected him in a manner most painful to behold. His face became distorted; the alæ of the nose worked convulsively; his lips moved quiveringly up and down; his eyelids were expanded into a wild and eager stare; the tongue was now stiff, now played convulsively within the mouth; and the muscles of the throat, larynx, and trachea were sympathetically affected. Thus, after terrible efforts, the boy gave utterance to a mangled and imperfect word;—now for a time was his speech free, and words chased one another with incredible velocity, till confusion ensued amidst the thronging sounds; and the same painful scene was thus again and again renewed. The peculiar physical horror which constitutes a stutterer, and which is excited by the effort to speak, is very similar to that which gives rise to the excitement and spasm of the hydrophobic patient at the sight of water. This internal movement might, on that account, be called phonophobia.

"The boy's mother caught eagerly at my offer to make an effort to cure him; accordingly, with the assistance of Drs. Holthoff and Hildebrandt, the operation was performed as follows: The boy sat with his head leaned against the breast of an assistant; the tongue being protruded as far as possible, was grasped on its anterior half with the forceps of Müzeux being thus compressed laterally, and drawn forwards by one assistant. The gentleman against whose breast the boy's head rested, retracted the angles of the mouth with a pair of blunt hooks. Grasping now the tongue as near to its root as possible, between the thumb and forefinger of the left hand, I passed the bistoury through it, and divided it completely from below upwards; a strong ligature passed through the posterior edge of the wound, served to fix it temporarily, and prevent too great a strain upon the slender band which alone connected the mass of the tongue to it; the anterior lip of the incision was now grasped, and laterally compressed between the modified hare-lip forceps, and a wedge-shaped slice excised out of the whole thickness of the tongue. It will be found more convenient to make this second incision from above downward, and with a small straight knife. The posterior edge of the wound was now, by means of the before-mentioned ligature, and a sharp double hook, drawn so far forwards that the needles with the ligatures could be conveniently passed through it; six strong sutures served to bring the edges of the wound together, and to restrain the hæmorrhage. To effect the latter object, they must include the whole depth of the wound within their loop. That the hæmorrhage was considerable, may be imagined from the nature of the operation, which should not be attempted by all persons indiscriminately. As soon as the boy's mouth was washed out, I desired him to pronounce some of those words which he had before found especially difficult; he did so without stuttering or hesitation. The distortion of the face, however, continued; the patient was put to bed, and a cooling plan of treatment ordered. With the exception of a slight sympathetic febrile disturbance, the swelling of the tongue, that one might anticipate, and the consequent impeded deglutition, nothing remains to be noticed, so far as regards his recovery from the operation itself. His features, and his mouth especially, were still much distorted when he spoke, but the stutter had entirely ceased. On the fifth day I removed three of the sutures; during the next twenty-four hours the swelling of the tongue had visibly decreased, and I then removed the three remaining sutures. On the seventh day the wound was completely healed the back part of the tongue alone was very inconsiderably swelled, and the boy quite reestablished. At this present time, not the slightest trace of stuttering remains, not the slightest vibration of the muscles of the face, not the most inconsiderable play of the lips. His speech is throughout clear, well-toned, even, and flowing. Neither inward emotions nor unexpected external impressions, produce the slightest hesitation; he can speak, read, and entertain himself indifferently with friends and strangers."

"The total number of stutterers that I have relieved up to this time is sixteen, and those who are as yet under treatment appear to promise equally favourable results."

The following paragraph is deserving of serious consideration, not less on the part of those afflicted with the serious inconvenience in question, but also by the operator. We are informed that in an operation for stuttering, performed a short time since in London, the hæmorrhage was fearful.

"In this operation it is more difficult to prescribe for the individual modifications of each particular case, than in the operation for strabismus, and it can never be performed by one who has not the temperament of an operator: the hæmorrhage must hold all others at a respectful distance. The extent and importance of the operation, the possible danger to life, or loss of the tongue, either through the want of skill in the assistants, who may tear it off when so nearly separated, or through mortification or ulceration of its connecting isthmus. These are contingencies rationally to be feared, and which must be carefully weighed beforehand."

Professor Dieffenbach concludes with a few words of criticism upon the herd of imitators who follow in the wake of talent, which are worth perusing, especially as they are evidently intended as a satire upon the instrument-discoverers, and new-method incubators of squint-cutting. Thus he observes,

"Amidst the prevailing rage for modifying operations, I foresee that my having described the three principal available methods, cannot fail to open to surgeons a vast field for the discovery of modifications, and the creation of instruments. We shall have conical and oblique incisions, from the surface and under the skin! Actual and potential cautery! We shall have knives and scissors with improved curves, and a thousand variously-fashioned forceps and hooks. They will set the blades at angles with the handles to allow of a better light falling into the mouth. Opportunity is likewise afforded to professional antiquaries to hunt after a name for this operation. To them I freely make over the right of baptism."

To this we may add a case reported by M. Franz, of London, who performed Dieffenbach's third operation, viz., the excision of a wedge from the root of the tongue on the first of March.

The patient (who was a young man aged 17) bore this severe operation well, but became rather faint towards the termination, and afterwards vomited large quantities of blood which he had swallowed. As soon as he had washed his mouth with a little water, I was exceedingly pleased to hear him pronounce words which, previously to the operation, he was utterly unable to articulate; such as time, powder, &c., without the slightest hesitation or stammering, and without any twitchings of the lips, or even convulsive movements of the muscles of the face or neck, and immediately afterwards I was surprised by his saying with facility and distinctness, "there is some blood running down my shirt." He was now put to bed, and desired to keep quiet, and directed not to be allowed to speak, and to have his mouth kept cool by means of cold water. On my calling in two or three hours time I found the case proceeding favourably; no reaction had as yet taken place.

March 9th.—Has for the first time taken a walk in the open air. The movements of the tongue less painful. The mother gives a favourable account of the progress of his speech.

Perhaps it may be as well to state that the muscles divided in this operation are the lingualis, the genio-hyoglossi, the hyo-glossi, and the stylo-glossi.

We here leave the subject without comment; experience alone can decide upon the merits, both positive and relative, of these different operations.

---

*Brief Notes on the Lake-Fever of Canada.* By Thomas Stratton, M. D., Navy Medical Department.—On the shores of Lakes Ontario and Erie, Lake-Fever is the popular name of intermittents and remittents. Farther back in the country they are generally called ague.

As the tide of immigration advances westward, these, the prevailing diseases of Canada, retire before it. Kingston, situated at the eastern extremity of Lake Ontario, is in 1840 very much healthier than it was in 1830; and Dunville, 200 miles, and Amherstburg 430 miles farther west, where now there are several cases of ague in every house in the course of the year, will probably in other ten or twenty years be as healthy as Kingston is now. Besides there being less fever at Kingston than at the other two places mentioned, its type is intermittent, while at Dunville and Amhertsburg it is often remittent.

Cleghorn, Fordyce, and some other writers, consider ague infectious; but, in numerous opportunittes I have had of inquiring into the accuracy of this observation, I have never noticed the disease to be propagated by infection or contagion.

The effect of sulphate of quinine, when not equal to stop the return of the paroxysm, is sometimes to make its accession an hour or so later, as in the following case.

Peter Heaton, aged 30, 1839, September 14th, had rigors at 1 P. M.; 15th at 2 P. M.; 16th, at 3 P. M.; 17th, at 4½ P. M.; 18th, at 5½ 19th, no paroxysm; 21st, cured.

In some cases the paroxysms occur at the same hour during the continuance of the disease, as will be seen in the following case, which also illustrates the premonitory yawning.

Wiliam Glover, aged 21, 1839—

| | | | |
|---|---|---|---|
| Sept. 12, | occasional yawning, | from 9 A.M. to 12 A.M.; | rigors at 12 A. M. |
| 13, | do. | do. | do. |
| 14, | do. | do. | do. |
| 15, | do. | do. | do. |
| 16, | do. | do. | do. |
| 17, | do. | do. | do. |
| 18, | do. | do. | 12½ A. M. |
| 19, | none | do. | none |
| 21, | cured | | |

The explanation of the yawning may be, that, while the cold stage is about to come on, there is congestion of blood in the internal parts, and among others in the brain, lessening a little its sensibility, and making it less sensible to the demand of the system for the continuance of the function of respiration. The premonitory yawning occurs in perhaps one-tenth of the cases.

Another symptom which often comes on an hour or two before the accession of the cold stage is coughing, which likely is from congestion of blood in the lungs.

The vomiting in the cold stage of the ague is often very severe; and I lately knew of a case where hernia was produced by it. When this symptom is very troublesome, I have often checked it by opium.

When the chilliness of the cold stage continues long, the patient derives great relief and comfort by taking a dose of from 10 to 15 grains of camphor.

In the hot stage, the symptom most distressing to the patient is severe headach. This I have often relieved or removed by having him removed into a strong current of air; at other times giving from 20 to 30 minims of laudanum, (as advised by Lind,) has afforded speedy relief. The opium has had this effect in every instance in which it has been used, in general sleep not being induced by it, merely removal of the headach.

In many instances the use of emetics is unnecessary, from the great vomiting which naturally occurs in the cold stage; and in other cases, I have not observed any particular good result from them;—many patients to whom emetics were not given recovering as soon as others who took several in the course of the disease.

A cure was found to be produced in the shortest time by administering calomel and compound extract of colocynth; and when three or four evacuations had been procured, giving a large dose of the sulphate of quinine, without much regard to the intermissions. Some recommend the quinine to be given in the intermissions only; others advise the whole quantity to be given in one dose immediately before the expected paroxysm; but as it is likely that the medicine requires an hour or two to produce any effect, its exhibition, in most cases, during the paroxysm, is not contraindicated, and by waiting for the intermission its effect upon the first subsequent paroxysm is rendered null.

Venesection in the cold stage was found by the late Dr. Mackintosh to shorten the cold stage, and break the habit of the disease. In four cases only in which I tried it, its effects were to alleviate and shorten the hot stage, the course of the disease not being stopped, and not appearing to be made shorter. During the last two and a-half years I have been in Canada, most of the people I have seen affected with ague have been spare-bodied and weakly; and if venesection in them were to fail in cutting short the disease, as it did in the four instances above-mentioned, it would most likely make the cure more tedious by adding to the patient's weakness. Many medical men in the country with whom I have conversed on the subject consider venesection in the cold stage objectionable in most cases, and in the remainder not to have the effect experienced by Dr. Mackintosh.

In the popular treatment of ague, much use is made of emetics (generally of mustard;) and the medicines with quack names, of which the people make use, are often, I believe, solutions of the sulphate of quinine.

When the patient is recovering there is often an eruption on the lips.

At Kingston, in ten months, July 1838—April 1839, in 150 men, there were seven cases of intermittent. I am informed that about ten or twelve years ago every third or fourth person in the neighbourhood was the subject of ague.

At Dunville and Amhertsburg, in ten months, May 1839—February 1840, of 70 men there were 51 attacked by marsh fever.

Kingston, north latitude 44°18′; west longitude 75° 41′: 234 feet above the level of the sea, has a population of 6000. The country round is hilly, mostly cultivated, and generally free from marshes. The Cataraqui bridge connects the town with a low peninsula, many hollow parts of which are below the level of the lake, and filled with water or dry, according as the water of the lake is high or low. The part of the peninsula alluded to is bounded by Navy Bay and Ereen Bay on two sides, and on other two by the "Common," and the hill on which Fort Henry is built. As at present vessels for the Rideau Canal pass through a part of the Cataraqui bridge to the interruption of passengers, there has been some talk of making a canal through the neck of the peninsula; and if this ever be done, the excavations from the canal will fill nearly all the hollows alluded to, and remove, in a great degree, the causes of what little lake-fever Kingston has still to complain. There is one and sometimes there are two regiments at this place, which is also a naval station.

Dunville, north latitude 42° 33′; west longitude 79°; 364 feet above the level of the sea, is on the left bank of the Grand River, five miles from where it flows into the eastern extremity of Lake Erie. The river is about fifty yards wide; the surrounding country thickly wooded, flat, and marshy; the banks of the river in many places scarcely a foot higher than the surface of the water. At Dunville there is a dam across the river for canal purposes, which has made the banks above the dam to be much overflowed, and also the banks below it, from the circumstance of certain winds making the water at the east end of the lake rise considerably, and flow up the river,—this reflex current, which before the existence of the dam extended eighteen or twenty miles up, being now stopped, five miles up, overflows the banks to a considerable extent. The residents in the neighbourhood are very subject to marsh fever, every family having several ill in the course of the season. The unhealthiness of this place will likely continue without any decrease, as it is not probable the dam across the river will be removed. This is a naval station.

Amhertsburg, north latitude 42° 36′; west longitude 82° 56′; 364 feet above the level of the sea; on the left bank of the river Detroit, one mile from where it flows into the western extremity of Lake Erie. The surrounding country is flat, and about half cleared of wood. Close to the town is a creek, which is filled chiefly by the overflowing of the river, and emptied by evaporation. By damming the mouths of this creek, where it is connected with the river and lake, and by draining, this marshiness might be removed; and this will no doubt be done in the course of ten or twenty years, when land shall become more valuable.

Ague and remittent fever are very common, most families having one member at a time ill during the summer and autumn. In the summer the heat is very oppressive. This is the most westerly British military station in America, and as it is the most southerly part of Canada, some may perhaps feel interested in an account of the temperature for twelve months.

| | | | | |
|---|---|---|---|---|
| 1839 | April range of thermometer, | | 38° to | 79° |
| | May, | do. | 46 | 84 |
| | June, | do. | 50 | 85 |
| | July, | do. | 61 | 84 |
| | August, | do. | 60 | 84 |
| | September, | do. | 47 | 78 |
| | October, | do. | 42 | 75 |
| | November, | do. | 2 | 60 |
| | December, | do. | 15 | 43 |
| 1840 | January, | do. | 5 | 45 |
| | February, | do. | 10 | 64 |
| | March, | do. | 23 | 60 |

It is not probable during the winter there can be a first attack of intermittent, as the marshes are sealed by the frost. Any case that occurs is most likely from exposure to wet in a person predisposed by having had the disease in summer.

I spent the winter of 1839—40 at Amhertsburg, and during it there were very few cases of marsh-fever; but on February 17th, there was a thaw, the ice over the muddy bank of the river floated away, and next morning several persons living in houses along the bank were attacked by ague. There is a regiment stationed here, and it is at times a naval station.

Sandwich is a small town, fourteen miles north from Amhertsburg, and similarly situated to that place as regards creeks, &c. Government has this year (1840) completed the erection of new barracks. There are two companies of a regiment stationed here; and I am informed that often one-half of the men at a time are ill of fever. The inhabitants are very suject to marsh-fever. This is occasionally a naval station.

Windsor is a small village, two miles north from Sandwich, and also on the river Detroit. The bank is here high, and there are no creeks as at the two towns last mentioned. New Barracks have been built here this year (1840,) which are at present occupied by militia. There is no ague here, though only two miles from a town where remittent fever is very general, and occasionally fatal. It is probable that the gaseous poison, which is the cause of intermittent fever, is not in a state of chemical union with the common air, but in a state of mixture only. Often in these marshes, early in the morning, I have observed a stratum of air two or three feet thick, resting on the ground, and of a darkish colour, and at the same time have felt a very offensive odour. These I have observed often iu the evening also, and then along with them a peculiar sensation of heat. These three phenomena have been noticed by persons walking with me, and who were not, like perhaps myself, looking for them.

Samia and Goderich, on Lake Huron, are very healthy localities. It is observed that militia regiments do not suffer so much from marsh-fever as regiments from Britain. In winter, as regards health, troops may be stationed at Sandwich with as much propriety as at Windsor. An objection to their being stationed in summer at Windsor is, that there is greater facility for desertion here than at Sandwich; but this advantage is gained by the loss cf the use of half the strength who are in hospital during the autumn.

*Kingston*, Upper Canada, Dec., 1840.

[*Edinburgh Med. and Surg. Journ.*

---

### EXTRACT FROM SIR B. BRODIE'S SPEECH AT THE ANNIVERSARY MEETING.

### *Eulogium on Sir Astley Cooper.*

Sir Astley Cooper was the fourth son of the Rev. Dr. Cooper, who was rector of Yarmouth, in Norfolk, and otherwise a gentleman of independent fortune. Dr. Cooper conducted the education of his sons in his own house, and Mr. Vince, who was afterwards well known as Professor of Mathematics in the University of Cambridge, was one of those who assisted him in this undertaking in the capacity of private tutor. It appears that his son Astley, at this period, displayed none of that disposition to industry which was afterwards so remarkable a feature in his character; being more distinguished as a lad of spirit and enterprise than he was for his love of learning. When he was about sixteen years of age an accidental circumstance occurred, which determined the lot of his future life. A boy was thrown from a wagon, and the accident happened in such a way as to cause a laceration of the femoral artery. Some other boys who were present were stricken with horror at the sight of a torrent of blood flowing from the wounded vessel, and ran away. Young Astley alone remained, and had sufficient presence of mind to tie a handkerchief round the thigh, so as to stop the hæmorrhage until the arrival of a more experienced artist. Enjoying the credit which he obtained by this exploit, he declared that he would be a surgeon; and his father, taking him at his word, sent him shortly afterwards to London, placing him under the late Mr. Cline as a private pupil. At that time of day a most absurd custom prevailed of sending boys to begin the study of surgery at the early age of fifteen or sixteen years; as if a good general education was of no value, and as if there was something in the surgical profession which none but a youth could learn. In young Astley's case the usual consequences ensued. His early studies were interrupted, and the first two or three years of his being in London were employed to no good purpose. It appears, however, that Mr. Cline discovered something in his idle pupil which led him to expect that he would turn out well ultimately; for, in the course of time, he gave him to understand that if he could qualify him for the task, he would give him the opportunity of beginning to teach anatomy as a demonstrator in the dissecting room of St. Thomas's Hospital. From this time the young man became an altered person, and his careless and desultory habits were exchanged for a degree of zeal and industry which could not be surpassed, and which he retained to the latest period of his existence.

In the year 1796, being then 28 years of age, with the sanction of his patron, Mr. Cline, he began to deliver lectures on surgery. He had previously attended those of Mr. John Hunter, and he conceived that he could follow in the steps of that great genius, whose object was to teach, not so much the details of practice, as what he conceived to be the principles of pathology and surgery. But he mistook his talent, and his class soon began to desert the theatre in which he lectured. This, however, only led to the display of another talent, for which he was remarkable during his whole life. No one could discover sooner than himself what he was fit for, and what he was not. He never allowed his vanity to mislead him on this point; and he never persisted in doing that for which he found that he was not well qualified by nature. On this occasion he was not wanting, and soon corrected the error which he had committed. He altered his system of instruction. He brought his notes of cases from the hospital, and communicated to his pupils the facts which he had collected and arranged. His discourses, full of important practical knowledge, became valuable and attractive: his class increased, and he soon became one of the most popular and influential lecturers of his day.

In the year 1800, his uncle having resigned the office of Surgeon to Guy's Hospital, he was elected to succeed him. He had now every opportunity of advancement which could be derived from circumstances, independent of his own character and conduct. His career as a hospital surgeon is well known to all of those whom I now address. You also know the important additions which he made to surgical literature, and the success which his very extended reputation obtained for him in private practice; and I feel that it would be idle for me to occupy your time with the details of facts with which you are already well acquainted, and which, in one way or another, have been brought so repeatedly before the public.

If it be generally interesting to study the character of those, who, by the exercise of their own talents, have raised themselves to a conspicuous station in society, it is so more especially on an occasion like the present, where the individual has been one with whom we have ourselves associated; whose early hopes and aspirations were similar to our own; who has been engaged in the same pursuits, and partaken of the same anxieties and gratifications with ourselves.

In reviewing the history of Sir Astley Cooper, the first thing that attracts our notice is the perfect devotion with which he followed the science of his profession from the very beginning to the very end of his career. No other pursuit stood in competition with it, and we cannot doubt that his attachment to it was genuine—that he loved it for its own sake, without reference to it as an instrument for amassing a fortune, or acquiring distinction or importance in the world. Some years ago, when his health was suffering from severe mental and bodily exertions continued during a long series of years, he retired to lead the life of a country gentlemen on his own estate, in Hertfordshire; but the experiment failed, and in less than a year he was compelled, by the want of interest in other objects, to return to his old employment. No alteration took place in this respect as he advanced in years, and the additions which he made to his anatomical museum, and his work on the structure of the female breast, bear honourable testimony to the zeal and activity of the latest period of his life. Zeal may exist without industry, as there may be industry where there is little zeal; but in Sir Astley Cooper these two qualities existed in combination, each tending to exalt the other. Even up to the time of his last illness he was in the habit of rising at half-past six o'clock in the morning, and was generally engaged in some anatomical or pathological dissections for two or three hours before he began what others would have regarded as the first business of the day. He was never without occupation, and having no desultory habits, he persevered steadily in any investigation that he had begun until he had completed it, as far as it was in his power to do so. A strong physical frame afforded him peculiar facilities of labour; yet, notwithstanding this, his exertions were at one period beyond his powers; and he informed me, not long since, that during a great part of the time when he was engaged in extensive practice, he was subject to attacks of vertigo. One of these, more severe than usual, occurred in the year 1824, attended with a slow and intermitting pulse. On one occasion his limbs gave way under him, and he fell to the ground. In the year 1827 he had a fever of an intermittent kind, attended with great general debility, and loss of power over the muscles of one arm, so that he could with difficulty raise any thing to his mouth. This alarmed him greatly; and seems to have been the immediate cause of his temporary retirement from his profession. He afterwards had a fit of the gout, which, combined with the repose of a country life, relieved his other symptoms.

It must be acknowledged that we find in Sir Astley Cooper but little of that inventive and discursive faculty which analyses, combines and abstracts; which perceives and realises remote relations and analogies; and to which we must attribute the exploits of genius, whether it be in poetry or science. It must also be acknowledged that, for the most part, he thought too rapidly for strict philosophical induction, and that he was apt to draw a general conclusion, even from a single instance, which nothing but an abundant evidence could justify. In truth, the construction of his mind was such that it was less fitted to deal with principles than it was to deal with facts; but in this last respect I have scarcely known his equal. He was a minute and accurate observer, and his perception of facts was so vivid, and the attention which he bestowed on them was so great, that almost every thing of any importance, which he had ever witnessed, remained indelibly impressed in his memory. Many who are now present can testify to the truth of this observation, and will well remember with what facility, when a remarkable case was presented to him, he could draw from the fund of his vast experience, describing some cases exactly like it which he had seen many years before, and others resembling it in some respects, and differing from it in others. He was scrupulously exact in all his statements, and no one could be more free than he was from that weakness to which even some honest persons are liable, who make themselves believe that facts are different from what they are, when it so happens that they do not precisely suit their preconceived opinions.

At one period of his life Sir Astley Cooper was much engaged in operative surgery, and an opinion has prevailed with many out of the medical profession, that skill in performing operations was the greatest talent which he possessed. Some of the absurd and vulgar, and, for the most part, unfounded anecdotes which have been published since his death, are calculated to give countenance to this opinion. But I need not tell the Fellows of this Society how great an injustice this is to the memory of our late associate. It is very probable that, as a young man, Sir Astley Cooper might have been highly gratified by the merit which he obtained as an operating surgeon, at a time when he had not yet had the opportunity of acquiring a higher and more substantial reputation. Many, however, have been his equals, and even his superiors, in this respect; and the estimation in which he was held among those who had a right to judge of his attainments, is to be attributed to his intimate knowledge of the history and progress of disease, and to his talent in selecting and applying the appropriate remedies: and to nothing else. The time may even arrive when it will be forgotten whether he was or was not expert in the performance of operations; but his public works will remain as long as surgery is cultivated, and especially the treatises on Hernia and Dislocations—replete with important facts, and containing the clearest rules of diagnosis—will transmit his name to posterity with those of Sydenham and Hunter, as a benefactor to the human race.

Sir Astley Cooper enjoyed an unusual degree of popularity for a long series of years, especially among the members of his own profession, and more than his character as a surgeon, however high it may have stood, can easily explain. He owed much of this to his moral qualities. He was considerate towards other practitioners; always ready to communicate whatever information he possessed in a simple and familiar manner; and he never attempted to obtain credit for himself at the expense of a fellow-labourer. In his intercourse with others he was essentially kind-hearted, and he possessed that degree of cheerfulness which made him acceptable to every one whom he met, and without which it is so difficult, and I may say impossible, to obtain an influence over the minds of other men. He possessed another great advantage in having no small degree of practical knowledge of human nature. By this I mean not that kind of knowledge to which this appellation is so frequently misapplied,—founded on a quick perception of the weak points of the minds of other men, by means of which individuals of small capacity are enabled to gain an ascendency over those whose intellects are superior to their own,—but a clear and ready insight into the whole of the human character, a knowledge of the qualities of the mind generally, and of the motives by which the actions of mankind are regulated.

There is one other point in Sir Astley Cooper's character which it occurs to me to notice before I quit the subject, and I do so merely because it gives me the opportunity of contradicting some most false and injurious statements which have been lately put forth respecting him. Although he derived from his practice a larger income than was ever obtained by any other person, either in the medical profession or in any other, he never exhibited in his profession any thing approaching to the vice of avarice. He was in the greatest degree liberal, and apparently careless as to the remunerations which he received. I say this with the utmost confidence, having never known nor heard of a single instance to the contrary during my long acquantance with him.—*Lond. Med. Gaz.*

---

*The Hospitals of Metz.*—The hospitals of Metz do not compose a whole, but preserve the distinctive stamp of their respective foundations, and are not united by that financial bond which constitutes one of the advantages or inconveniences of centralization. The congregation of which St. Vincent de Paul was the creator, and is still the life and soul, manages three *hospices*, namely, the charity and orphan-house, and the hospitals called *Bon Secours* and St. Nicholas. The last one is of a mixed kind, and resembles those which in most of our great provincial towns are called general hospitals, as at Montpelier, Toulouse, &c. St. Nicholas' hospital is at once a retreat for indigent old age, and a medical asylum for the scrofulous, the epileptic, and different incurable affections; foundlings and deserted children are also admitted there. In spite of the order which reigns in this mansion of so many guests, it is impossible to approve of such an arrangement. It is not at Metz alone that we have to lament the extent of such an institution; these immense hospitals are a piece of municipal vanity, when they are not the unwholesome result of buildings successively heaped together by economy or routine.

The *Bon Secours* is the real hospital of Metz, the clinical theatre which from year to year, from season to season, displays the phases of local disease; it is there alone that there is a succession of patients, and that observation can be exercised on fresh objects; but we are sorry to say that it is far from being an institution which should have had the double object of liberal charity, and the progress of the art of healing. It is situated near the ramparts, in a part of the town where the indigent are crowded together; its back looks on the stream of the Moselle, from which it is separated only by a small garden; nor does its internal arrangement compensate for the badness of its position. It cannot hold more than 140 to 160 beds. When I visited the hospital, however, with my friend, Professor Forget, who was then going the circuit at Metz, as president of medical juries, the number of beds was more than enough, as there were not more than fifty patients in the house; but we learned from the superior sister, that at certain periods of the year, disease increases so suddenly, that it is necessary to refuse admission to a great number of applicants, who are obliged to content themselves with being visited at home, and receiving medicines gratuitously. There are two large wards on the ground floor; they are paved, and it must be difficult to warm them in winter. There is the same arrangement on the next floor, with some additions. Fresh air is easily introduced, and one observes that exquisite cleanliness (*cette coquetterie de lustre et de propreté*) which distinguishes houses entrusted to the rule of female corporations. All the wards are not yet supplied with iron bedsteads, and the space between the beds did not seem to us in proportion to the mass of atmosphere; this inconvenience is increased by enormous pillars, which might be usefully replaced, as at the Val-de-Grâce, by thin cast-iron columns, the slim and elegant supporters of the upper story. In going over the wards we saw venesection performed by one of the sisters; and we learned, not without surprise, that the hospital has neither in-door nor out-door dressers, and that that the dressings, as well as the small operations of surgery, are executed by the sisters. There is no house-surgeon; no apothecary; the shop also belongs to the sisters; one of them presides over the composition of diet-drinks, and the preparation of the daily prescriptions. The hospital receives the chief medicines and chemical products from a druggist in the town; and a dexterous and exact sister weighs, doses, combines, mixes, triturates, dissolves, edulcorates, and adds the usual ornament of a label to the daily series of her elaborations. It would be impossible for us not to do justice to the symmetry of the internal arrangements of the laboratory, the cleanliness of the pots and utensils, the brilliant polish of the scales, the spotless white of the linen in the wards, the affectionate attention of the sisters to the patients, and the quickness of their movements round beds where patients were to be dressed; but we confess that all this was unable to reconcile us to a hospital without in or out-door pupils, without apothecaries, without any scientific impulse in the details, without the steady control of a superior jurisdiction. These offices ought not to be confided to female hands: give a house-surgeon to these patients, among whom Science, like the deity whom their pain invokes, ought to be always present, always attentive; open these wards to the intelligent observation of pupils, who, at a later period, after having received the doctoral stamp, will be to Metz practitioners early familiarized with the morbid forms of the place, and the genius of indigenous disease. The sisters, moreover, have the exclusive right of walking in the only garden of the hospital; it is clear that the patients ought to be admitted to this privilege. We diligently inquired of the superior what was the diet of the house, and both M. Forget and I were convinced that it is insufficient for convalescents, who should be nourished as well by the quality as the quantity of food. This defect, and many others, (such as the want of particular clothing for the patients,) arise from the scantiness of the funds at the command of the governors; indeed, the annual budget of the town will not allow of a complete remedy; yet it cannot be doubted that the more direct and decisive intervention of medical authority in these hospitals would correct some of the errors which prevail there.

The *Bon Secours* is appropriated to town patients; those from the department are admitted only when the communes to which they belong pay for their maintenance. A sad necessity! The gates of a hospital ought to open to suffering, from whatever village or market-town it may have dragged its slow length along. Why ask for a certificate of birth and origin from the man who is about to die? Does charity know the limits of departments, stamp its deeds with the seal of a commune, or select its harvest on a privileged soil? Our fathers built the house of the sick by the side of the house of the Lord, and they called it the Hôtel Dieu, consecrating it as a pious and helpful hostelry for all the pains which wend their way, lame and crooked, over this earth. As the gate of the church is open to every devotion, that of the hospital ought to yield to each languid hand which knocks at it. Governors and physicians, suffer all the sick to come to you, as our Divine Master suffered all children: do not the ailing and the weak compose the perpetual childhood of humanity?

A thought occurred to us at Metz which has already been expressed in this journal, and which our pen will never be tired of repeating; a thought which contains in itself a host of reforms and improvements. It is simply this:—let the government take into its hands all the property and income belonging to all the hospitals and *hospices* in France; and let every department have its share in proportion to its general population and its annual contingent of sick poor; and let all these establishments form part of a general system, governed by a uniform statute vivified by a homogeneous administration, and let it be under the parliamentary control of the Chambers.

Once more we announce this wish, which we number among the desiderata of each new year; may it germinate, and fructify; may it one day ascend the tribune, in the person of some honourable man imbued with the real interests of the country; and may a minister, capable of occupying himself with something besides the preservation of his portfolio, embody it in the form of a bill! While waiting for this miracle, let us finish our rapid glance over the medical institutions of Metz.

One of the most useful is the practical school of midwifery of the department. It is under the direction of Professor Morlanne, who, with two colleagues, bears the burden of teaching. There is a course of midwifery, and a *clinique* for the diseases of children, and of women in childbed; and gratuitous advice is given daily to town and country patients. This advantage seems to be appreciated, for last year no less than 1423 persons applied for advice. The *Hospice de la Maternité* is distinct from the Ecole d'Accouchement. It was founded in 1334, by a citizen of Metz, named Jean La Hungre, for poor pregnant women; and the deed was confirmed in 1337, by the bulls of Pope Clement VI. The sisters of St. Felicity attend in the house, and, which seems strange for nuns, are both midwives and nurses.

Besides these public institutions, Metz affords other relief to the poor. The Sisters of St. Christian, whose chief residence is in this town, and who pass their noviciate here, devote themselves to the sick, and to the instruction of the poor. Their assistance is sought by the sick of every sect, which is the finest panegyric that can be given them.

The municipal board of charity has divided the town into five sections, and gratuitous advice with home visiting are to be found in each. With an honourable solicitude, the physicians of Metz share among them this domain, where science meets religion in order to bring living streams into a withered land, and in the same beings to revivify the body which decays, and the soul which degenerates.

There is an Israelite Hospital, established by a royal ordinance of May 5th, 1832; it contains only eleven beds.

The midwifery school is of secondary rank, and is intended for midwives alone.

For science, there is a military hospital of instruction, and a medical society. The latter participates in the lot of all provincial academical associations; there are alternations of languor and life; and years of silence and death are followed by a sign of life in the shape of a volume. The Metz society has lately published a volume, and I arrived just in time to receive it from the skilful and learned Dr. Maréchal. Some day I shall return to it, as it contains several interesting papers; in particular, a very good report on the epidemic cholera at Metz. The book has a tendency to usefulness which I will not pass over, and is especially occupied with questions of public hygiene as applied to the city of Metz. This society has been engaged with the useful, while so many others are like the astrologer in the fable.

The hospital of instruction is, without doubt, the true representative of the medical science of Metz, from the scale on which it teaches, from the merit of the men who have successively directed it, from the extent of the practical theatre which the art possesses there, and from the influence which it exercises over a body of pupils frequently renewed, and many of whom have taken a distinguished rank in civil and military medicine. The hospital of instruction has, moreover, had the honourable privilege of furnishing the town with its favourite practitioners. There is not an inhabitant of Metz who does not pronounce with respect and gratitude the names of Gorsy, Marchand, Rampon, Moizin, and Wuillaume; the military hospital has supplied the Institute with an eminent chemist—Serullas; and has given two inspectors to the army medical board—MM. Moizin and Brault. M. Bégin, now first surgeon at the Val-de-Grâce, belonged to this hard-working school; Boisseau, author of the "Pyrétologie Physiologique," died there; it has just sent M. Scoulteten, author of the *méthode ovarlaire*, to the Strasburgh Hospital; and a number of other remarkable persons belonging to the Metz hospital of instruction. The distinguished men who now conduct it are unable to reap all the fruit of this long-enduring reputation, or to maintain the dignity of the institution against the injustice of the military spirit which rules at Metz, and casts its shade over every sort of merit which is not expressed by a big epaulette. Fortunately, science escapes from garrison influence, and moves at ease in a vast and handsome establishment capable of holding 900 patients; and fortunately, the *officiers de sante* of the army, and the body of teachers at their head, have never sought for the springs of their emulation and their zeal anywhere but in themselves.*—*Ibid.*

---

*On Distortions of the Chest and Spine in Children, from Enlargement of the Abdomen.* By JOHN SNOW, M.R.C.S.†—It is not my intention to describe the various deformities to which the chest and spine of children are liable, but only to speak of one or two distortions which arise from enlargement of the abdomen—a cause of deformity which, hitherto, has not, that I can find, been recognised by authors.

I shall relate only one, out of a few cases that I have witnessed, which will serve sufficiently to illustrate the points that I wish to establish.

Aug. 16, 1839. Hugh Lynch, a twin child, aged two years and five months, has had a double scrotal hernia from birth. He has been ill for the last four months; his mother says much in his present condition. The abdomen is very large and tympanitic; the chest is broad behind, very narrow in front, and flattened at the sides; the sternum projects forwards very much, especially at the lower end. The cartilages of the ribs, instead of passing outwards from the sternum on each side, leave that bone at an angle by no means very obtuse, and pass backwards to meet the osseous part of the ribs at another angle; and the cartilages of the false ribs project laterally from the bony portion, so that the lower part of the chest is much expanded where it unites with the enlarged abdomen. The last of the dorsal and first of the lumbar vertebræ project backwards, whilst the lower lumbar vertebræ project forwards and the sacrum backwards. The child is emaciated and feverish; its bowels are disordered, and its appetite is craving. Its mother feeds it chiefly on potatoes. The breathing is quick, the inspiration being easy, but the expiration difficult, and attended with an effort approaching to a cough; the upper part of the air-passages being closed after each inspiration, and then the air escaping with a slight sound, similar to that which takes place after the breath has been held for the performance of any muscular exertion. During each inspiration the abdomen descends and protrudes, and the cartilages of the ribs are forced inwards on each side of the sternum. During expiration, on the contrary, the abdomen retreats, and the ribs return to their previous situation. There is loud puerile respiratory murmur, and the chest yields a clear sound on percussion at all parts.

To take some Hydrargyrum cum Creta, and be fed in a more rational manner.

Sept. 27. The child is more emaciated. His belly is still large, but much less than before. His chest is of the same form, but the cartilages of the true ribs do not fall in so much during inspiration; those of the false ribs, however, are drawn inwards by the diaphragm at each inspiration, and so project towards the lower end of the sternum, whilst a hollow is left just beneath at the scrobiculus cordis. The thorax yields a clear sound on percussion, but there is a mucous rale.

The child died on Oct. 29th.

*Examination, seven hours after death.*—The spine is now pretty straight. Abdomen very much less than formerly, but yet tumid. The chest yields a dull sound on percussion throughout the greater part of its extent, although a few hours before death it sounded clear. On opening the abdomen, the diaphragm, instead of its usual arched form, is found to be stretched horizontally across the body, so that it is not so high as the seventh rib. The lungs are healthy in structure, but the whole of the left lung and the lower lobe of the right are collapsed, and totally void of air, and gorged with dark fluid blood; the remainder of the right lung is crepitant and healthy. The heart is healthy, but the pericardium contains three or four drachms of serum. Each rib is enlarged into a spongy head at the part where it unites with its cartilage.

The large intestines are distended with flatus, except in portions where they are firmly and preternaturally contracted. The colon is so much lengthened that it crosses the abdomen three or four times. The cœcum is in the usual position, and from this the colon extends to the left side and back again, then passes upwards to the stomach, across the abdomen, and down the left side in the usual route to the sigmoid flexure; which flexure extends into the right iliac fossa, and back to the median line, where it unites with the rectum. The remainder of the abdominal viscera were healthy. The head was not examined.

In order to show satisfactorily that this deformity of the chest is caused by the enlargement of the abdomen, I must prove that the space within the thorax is increased by any great distension of the abdomen, and not diminished, as is generally supposed. As the belly increases in size, the false ribs, with their cartilages, are pressed upwards, and approaching to a right angle with the spine; the circumference of the chest is thus increased, and the abdominal muscles, which by drawing down the ribs are the chief agents of expiration, can but ill perform their dury; they are kept on the stretch by the bulging out of the viscera, or whatever they enclose. Moreover, the diaphragm, being attached to the base of the chest all around, has its borders drawn further apart by the increased circumference of the thorax, and thus its natural arched form is removed; it approaches to a plane partition, and the chest, so far from being encroached upon by the abdominal cavity in this direction, has its perpendicular length increased.

---

* Abridged from an article by M. Michel Levy, in the Gazette Medicale of February 6, 1841.

† Read at the Westminster Medical Society, on March 13, 1841.

I may here state, I have always observed that the difficulty of breathing arising from enlarged abdomen, whether in human beings or quadrupeds, consisted in obstructed expiration, and not obstructed inspiration, when there was no other cause of dyspnœa.

The bellies of children are subject to a very much greater proportional enlargement than ever obtains in the adult, and the cartilages and ligaments of the ribs being more flexible and distensible, the expansion of the base of the chest becomes very great; and as the lungs are compelled by the atmospheric pressure to occupy every part of the chest, they must either be preternarurally distended, or the chest must be depressed in some other direction. Now, as the ribs and their cartilages are slender and flexible in children, the latter takes place; the chest becomes depressed laterally, and the sternum projected forwards, whilst a channel is left down each side of the chest where the cartilages unite to the bony portion of the ribs. The action of the diaphragm, which presses down the abdomen, and at the same time draws up the cartilages of the lower ribs, during each inspiration, makes room for more air than the lungs are inclined to receive, and the sides of the chest are pressed further in during each inspiration, and return again during expiration: thus the motion of the ribs becomes the reverse of the natural one. I do not conceive this arises from the mechanical resistance of the lungs, but from the sudden stoppage at the throat to the further access of air, which I have observed to be never absent during this deformity with inverted motion of the ribs. It is made evident by the slight explosive sound when the passage is again opened at each expiration. This check to the further ingress of air is no doubt a voluntary or instinctive effort to avoid the uneasy sensation arising from too great distension of the lungs. I do not think it consists in a closure of the glottis, but in the approximation of the posterior palatine arches, and the pressure of the root of the tongue at the same time against the palate of the mouth: the method by which, as Dzondi has discovered, the breath can be held. It is the office of the serratus magnus and the pectoral muscles to expand the sides of the chest during inspiration; but the diaphragm enlarging the chest more powerfully in another direction, these muscles yield to the atmospheric pressure, and eventually, so far as respiration is concerned, become paralysed.

I have never seen enlargement of the abdomen to great extent in a child under three years of age, that was not accompanied with this deformity of the chest. The degree of deformity is always in proportion to the enlargement; and in observing any individual case, the deformity is found to increase with the increasing size of the abdomen. Even when the enlargement of the belly is not great, there is a tendency to this deformity observable in the slight lateral projection of the cartilages of the false ribs. After the age of three or four years, I have not observed this deformity to commence, probably because the ribs become of a strength which prevents it; besides that the abdomen is not so liable to become tumid.

The other deformity, that of the spine, seems to be only an occasional, and not a constant attendant on enlarged abdomen: I think it is in the worst cases that it prevails. It consists in a projecture, frequently an angular one, of the last dorsal, or great lumber vertebræ, or both. In the case I have just detailed, it was accompanied by a secondary projecture of the sacrum; and in another case it was attended with a slight lateral deviation. This projecture of the spine is probably caused by the stretched abdominal muscles and integuments, drawing, by means of the pelvis and chest, on the opposite ends of the spinal column, whilst the increased contents of the abdomen make a resistance in the centre. That this angular projecture of the spine depends on disease of the bodies of the vertebræ in the first instance, we are forbid to suppose, by the fact that the projecture subsides as the abdomen diminishes.

In one child, an angular projecture for which the formation of issues had been previously recommended, perfectly disappeared as the child resumed his health, under tonic and alterative medicines, with attention to diet, and bandaging of the abdomen. It most likely depends on partial absorption of the intervertebral substance, or bending of the bodies of the vertebræ at the affected part, and, if continued, might no doubt lead to permanent disease.

Baron Dupuytren, in a paper published in the "Repertoire Géneralé d'Anatomie et de Physiologie," in 1828, described a deformity of the chest of children, which I believe, in many of his cases at least, to be the one of which I am speaking. It occurred in children badly clothed and fed, born of unhealthy parents, and living in damp situations, and was accompanied, in most cases, by enlarged tonsils, requiring sometimes to be extirpated. He said that there was a heel-like projecture of the sternum in front, and a sharp prominence of the spine; that the ribs were not only flattened, but that they were sunk into the chest as if they had been compressed from one side towards the other. This deformity was accompanied with great difficulty of breathing. He did not mention the abdomen, except in his preliminary remarks, to say that there was a projecture forwards of the sternum and belly; and he only alluded to it, I think, in one of his illustrative cases, in which, however, he said the belly was five times as large as the chest. In another case he spoke of the width of the chest at the base. He appeared to attribute the deformity to arrest of ossification, and softness of the bones; and recommended some mechanical measures and exercises, in addition to medical treatment, for its cure. He spoke of some cases in new-born infants, which, I think, could not have the same origin as the cases I have seen. The nature of the connection between these deformities and the enlargement of the tonsils, he could not tell.

I have only seen one case since I read Baron Dupuytren's paper, and in this there was no enlargement of the tonsils. I can, however, suppose that the obstruction to inspiration from enlarged tonsils, might cause the ribs of a child to be pressed inwards by the atmosphere, and produce a deformity of this kind.

In the Medical Gazette of January 12th, 1839, is a letter by Mr. Reese, describing the deformity of the chest of which we are treating as occurring in four or five children seen by him at the Tower Hamlets Dispensary. He describes the depression at the line of union between the ribs and their cartilages, producing a channelled appearance external to the sternum on each side; and he likewise describes the inverted action of the ribs in respiration. Mr. Rees does not mention the abdomen, except in the case he relates for illustration, in which he incidentally says it was tumid. He attributed the deformity to chronic pneumonia, which causing the lungs to shrink and become solidified, the ribs are forced in by the atmospheric pressure to occupy the space. We can perceive that shrinking of the lungs might have this effect, but in the cases I have witnessed there was no disease of consequence in the lungs, except in one child that had hooping-cough.

Mr. Amesbury describes this deformity, and the enlargement of the abdomen in connection with it; but he does not speak of them in the relation of cause and effect. He attributes this deformity to weakness of the muscles. He says, "Deformity of the chest, arising from weakness of the muscles, commonly takes place during teething, but may be produced by any complaint that tends to debilitate the system. This distortion usually assumes the form called 'chicken-breast.' In this variety of deformity the chest is usually more or less contracted laterally, the sternum is thrust forward, and the abdomen is preternaturally enlarged. The intercostal muscles act very little in respiration, the breathing being principally abdominal." Together with medical treatment for the improvement of the general health, Mr. Amesbury recommends bandaging the abdomen for the cure of this deformity.

Baron Dupuytren and Mr. Amesbury do not mention the reversed action of the ribs in breathing. Mr. Rees and Mr. Amesbury do not allude to any deformity of the spine; and the projecture to which Baron Dupuytren alludes appears to be one of the whole spine, and not of particular vertebræ.

In two or three of the cases I have seen, the inferior extremities became deformed under the weight of the enlarged belly, but it was the joints which yielded, and not the shafts of the bones; and there was only in one case the enlargement of the heads of the bones peculiar to rickets: where rickets exists it may assist to aggravate the deformity, but cannot of itself cause it. A scrofulous diathesis, by predisposing to mesenteric disease, may be considered favourable to the development of these deformities; but in the cases I have seen, I do not remember to have observed enlarged glands, or other decided marks of scrofula. And in the only two cases in which I have had the opportunity of an examination after death, there was no disease of the mesenteric glands, and the enlargement depended chiefly on the elongation and distension of the colon.

I have generally been able to trace the increased size of the belly to improper food, and have found it mostly among those infants who, from the poverty or intemperance of their parents, are, after weaning, fed almost entirely on potatoes. I believe the best treatment to consist in alteratives and tonics, with occasional purges, and the careful avoidance of crude and indigestible food. At the same time the abdomen should be firmly bandaged, as recommended by Mr. Amesbury, which measure, while it assists to reduce the belly to its natural size, will relieve the breathing by aiding the efforts of expiration; and by pressing the diaphragm upwards, will reduce the capacity of the base of the chest, and thus lessen the cause of the contraction higher up. If this deformity be left to itself, and should not prove fatal, the serratus magnus, and other muscles, may not recover their power of expanding the ribs, or the ribs may have become firm in their abnormal shape, and the deformity may continue, after the enlargement of the abdomen, which gave rise to it, has subsided. I have seen two or three cases of this deformity in grown-up persons, which, so far as I could gather the history, appear to have originated in this manner.

Thus, then, I have endeavoured to establish that enlargement of the abdomen in children leads to deformity of the chest, and occasionslly of the spine; and that although the deformity of the chest, or one nearly resembling it, may now and then arise from other causes, yet that this enlargement is of itself sufficient, and will never fail to induce it, if proceeding to any great extent in young children.—*Ibid.*

---

*A Case of Slow Pulse, with Fainting Fits, which first came on two years after an injury of the neck from a fall, and proved fatal five years and three month after the accident.* By T. H. Holberton, of Hampton, Surgeon Extraordinary to the Queen Dowager.—The subject of this case was a gentleman, 64 years of age, who received an obscure injury to the neck from a fall from his horse, in December 1834. From this injury he slowly recovered, and at the end of a year complained only of some difficulty in moving his head. Nothing further was remarked respecting this gentleman until January 1837, when he had a fainting fit, and Mr. Jackson, of Stamford, then observed for the first time that his pulse beat only 20 in a minute. From this time he had similar attacks, at irregular intervals, and from various causes, and in the spring of the same year, from excitement at a horse-race and long fasting; and another in the following June from a somewhat similar cause. In the latter instance, his pulse was 25 in a minute. When the author first saw him, in March 1837, his ordinary pulse was 33; but it was easily altered. Mental excitement usually increased it; and, in general, this was followed by a corresponding slowness of the pulse, and often by a fainting fit. The general characters of the pulse, when the patient felt well, were firmness, fulness, and freedom; generally it was regular, but sometimes intermittent. An overloaded state of the stomach was an occasional cause of these attacks; and in 1838, when the patient suffered from "a severe alarming succession of fits," the pulse fell to 12, 10, 9, 8, and, at three or four different times, was counted as low as 7½ in a minute. One of these attacks ended in the death of the patient, in April 1840.

The examination of the body was made by Mr. Liston, from whose report the following is extracted:—"The medulla oblongata was small in size, and extremely firm in consistence; the foramen magnum was altered in shape, the antero-posterior diameter being much diminished; the superior part of the odontoid process of the axis appeared to have been pushed back, and somewhat raised above its usual situation; the antero-posterior diameter was so much narrowed, that it would not admit of the little finger; the dura mater and ligament covering the posterior part of the body of the axis were much thickened; the atlas was in its usual situation, but the articular cavities were firmly ossified to the condyles of the occipital bone, and permitted no motion whatever between the atlass and skull." The patient never had paralysis, nor alkalescent urine; neither did he suffer from pain in the neck after the first few weeks from the accident.—*Trans. Royal Med. and Chirurg. Soc., in Lond. Med. Gaz.*

---

*Case of Neuralgia.* By T. T. Lambert, Member of the College of Surgeons.—E. L., a female, aged sixty-three, residing in Osborne street, had for several years been affected with a most painful condition of the nerves of one side of the face, coming on in paroxysms of the utmost severity, mostly towards night. At various times she had tried most of the usual remedies, without experiencing any thing but temporary alleviation. On the 8th of November, during the night, I was sent for, and found the patient in a state of the severest pain. I administered a powerful opiate, which did not produce any material relief; and sleep was not procured until the system was completely exhausted and worn out by suffering. We had the same painful scene repeated on the following night. Under these circumstances, as the branches of the facial nerve seemed to be principally affected, the pain shooting across the cheek down towards the lower jaw, and occasionally upwards towards the temple, I proposed the division of the facial nerve in the parotid gland, after its exit from the foramen. To this the poor woman gladly consented, and I proceeded to perform the operation by making an incision betwixt the mastoid process and the lobe of the ear. My next incision brought me in contact with a nerve, which I supposed, from its situation, to be the auriculus magnus nerve, one of the ascending branches of the cervical plexus. On raising this nerve with my forceps, a state of tension seemed to be produced upon all the nerves of the face which were affected with the pain, and the patient exclaimed that was the place from whence all her pain proceeded. Under these circumstances, as the operation for the division of this nerve is trivial compared with the division of the facial, and as it was just at hand, I determined to try the effects of its division; and the result was most satisfactory: the woman passed a delightful night, and enjoyed some good sleep. The next day she had little pain, and from that time to the present, she has been perfectly well, except having, on one occasion, a very trifling degree of pain. Four months have now elapsed since the performance of this operation, and during this interval the patient has been exposed to the intense inclemency of the recent winter. I am not one of those who would pay too much attention to a solitary case; yet, as a single fact, this seems to me to be important, inasmuch as the means employed have effectually cured, for four months at least, and I hope permanently, a very severe case of neuralgic pain. If I may be permitted to draw any pathological inferences from the fact, I think they would be the following. First, that some of the most severe forms of neuralgic pain are dependent not upon any diseased condition, but simply upon their possessing too great a quantity of their proper nervous energy; and secondly, that this can be reduced by cutting off a collateral, as well as a direct channel of supply.—*London Med. Gazette.*

---

*Internal abscess opening into the intestinal canal.*—A countrywoman, aged thirty, four days after recovering from an attack of sporadic cholera, was seized with suppression of urine, and pain in the abdomen, which were followed by a swelling between the umbilicus and the symphysis pubis, a little towards the left side. The case was terminated, and a cure effected, by evacuations of fœtid yellow pus by stool.—*Ibid., from Danish Medical Reports.*

